# Prenatal family income, but not parental education, is associated with resting brain activity in 1-month-old infants

**DOI:** 10.1038/s41598-024-64498-3

**Published:** 2024-06-13

**Authors:** Aislinn Sandre, Sonya V. Troller-Renfree, Melissa A. Giebler, Jerrold S. Meyer, Kimberly G. Noble

**Affiliations:** 1 Department of Biobehavioral Sciences, Teachers College, Columbia University, 525W 120th Street, Russell Hall 21, New York, NY 10027 USA; 2 Department of Psychological and Brain Sciences, University of Massachusetts Amherst, Amherst, MA 01003 USA

**Keywords:** Socioeconomic status, Family income, Parental education, Electroencephalography (EEG), Resting brain activity, Infancy, Neuronal development, Human behaviour, Language

## Abstract

Childhood socioeconomic disadvantage is associated with disparities in development and health, possibly through adaptations in children’s brain function. However, it is not clear how early in development such neural adaptations might emerge. This study examined whether prenatal family socioeconomic status, operationalized as family income and average years of parental education, prospectively predicts individual differences in infant resting electroencephalography (EEG; theta, alpha, beta, and gamma power) at approximately 1 month of age (N = 160). Infants of mothers reporting lower family income showed more lower-frequency (theta) and less higher-frequency (beta and gamma) power. These associations held when adjusting for other prenatal and postnatal experiences, as well as infant demographic and health-related factors. In contrast, parental education was not significantly associated with infant EEG power in any frequency band. These data suggest that lower prenatal family income is associated with developmental differences in brain function that are detectable within the first month of life.

## Introduction

Family socioeconomic status (SES)—commonly operationalized as parental educational attainment, occupational prestige, and family income—is a powerful predictor of children’s development and health^1^. Children who grow up in families facing socioeconomic disadvantage tend to score lower on assessments of cognition, language, and socio-emotional development, and are at higher risk for psychopathology across their lifetime^2–4^. Such disparities emerge in infancy and widen over time^5,6^, underscoring the importance of early prevention and intervention. However, the mechanisms through which family SES contributes to disparities in early childhood development are unclear.

One way that socioeconomic disparities might contribute to such differences is through adaptations in the normative development of brain function^7,8^. The brain undergoes significant change and is particularly sensitive to environmental influence during the first year of life^9,10^. Family SES is associated with variation in environmental exposures and experiences that may shape developing brain function^7,11^. However, it is unclear how early in life such differences emerge. A better understanding of the developmental time course of socioeconomic disparities in brain activity may inform the timing and design of preventative interventions and policies that support children’s long-term development and health.

Electroencephalography (EEG) provides a direct, non-invasive measure of electrical brain activity, which is useful for understanding socioeconomic differences in infant brain function^8^. EEG captures network-level oscillations in brain activity at the scalp surface and is commonly measured at rest in infants. Resting EEG is indexed along two primary dimensions: frequency and power. “Frequency” refers to oscillatory brain activity that occurs throughout the brain at different rates and is quantified across canonical frequency bands. Some bands represent lower-frequency (slower) oscillations (e.g., theta) and some represent higher-frequency (faster) oscillations (e.g., alpha, beta, and gamma). “Power” refers to the amount of brain activity in a particular frequency band; more EEG power reflects greater electrical activity generated by the brain.

Studies suggest noteworthy developmental change in lower- and higher-frequency EEG power across early childhood. In typically developing children, lower-frequency (theta) power decreases while higher-frequency (alpha, beta, gamma) power increases from infancy to middle childhood^12,13^, reflecting cortical maturation and increasingly efficient neural organization^12^. Variation in lower- and higher-frequency power is also associated with infant’s concurrent functioning, with more higher-frequency power linked to higher language^14^, cognitive^15^, and socioemotional scores^16^ and more lower-frequency power linked to behavioral, attention, and learning problems^17^. Additionally, more lower-frequency and less higher-frequency power in infants predicts lower language scores^18^ and increased risk for psychopathology in childhood^19^. EEG power may thus serve as a promising biomarker of future developmental trajectories.

Family SES has been associated with developmental differences in lower- and higher- frequency brain activity within the first few years of life. Infants exposed to family socioeconomic disadvantage tend to show more lower-frequency (theta) and less higher-frequency (alpha, beta, and gamma) power compared to infants from more advantaged circumstances^20–25^. Similar patterns of brain activity have also been found in infants facing other forms of deprivation^26^, and these differences may persist throughout childhood and early adolescence^20,27^. Further, boosting family income through unconditional cash transfers to families facing poverty may cause increased higher-frequency power in infants^28^.

Despite evidence linking family SES to differences in infant brain activity, it remains unclear how early such differences begin to emerge. For instance, some studies suggest that family SES is not associated with brain activity during sleep in neonates (12–96 h after birth^29^), while others have observed associations in awake 2–3-month-old infants^25,30^. Combined, these studies suggest the need for further research examining associations between family SES and brain activity in infants shortly after birth.

Additionally, family SES is a multi-dimensional construct comprising correlated yet independent factors^31^, and these factors may show different associations with infant brain function. Yet, few studies have simultaneously considered family income and parental education^22,23,30^, making it difficult to identify common and distinct associations.

This study aimed to examine whether family SES during the prenatal period prospectively predicts variation in infant brain activity approximately 1 month after birth. Specifically, we examined whether prenatal family income-to-needs and years of parental education are associated with infant EEG power in four frequency bands: theta, alpha, beta, and gamma. We hypothesized that higher prenatal family income-to-needs and higher parental education would be associated with proportionally less lower-frequency power (theta) and proportionally more higher-frequency power (alpha, beta, gamma) in infants. We also conducted exploratory analyses to examine whether associations between prenatal socioeconomic factors and EEG power were stronger over certain regions of the scalp (i.e., frontal, central, parietal, temporal, occipital). Finally, given that differences in infant brain activity may stem from early experiences that covary with SES^11^, we tested whether any significant associations between prenatal family SES and infant EEG power held when adjusting for various prenatal and postnatal experiences, infant demographics, and health-related factors.

## Method

### Participants

Mothers were recruited from local prenatal clinics, community events, and social media to participate in a longitudinal study examining associations between early experiences and child development. Participants were from the New York City metropolitan area and were intentionally recruited to span a range of educational attainment, from having less than a high school education to holding an advanced degree. Mothers were recruited over two time periods because of a temporary interruption in data collection due to the COVID-19 pandemic: the first cohort of mothers was recruited from June 2019 through March 2020 (N = 93) and the second cohort of mothers was recruited from August 2021 to September 2022 (N = 116). Mothers were screened over the phone to confirm eligibility. To participate, mothers were required to be 18 years of age or older, at least 35 weeks pregnant, carrying a singleton fetus with no known neurological or developmental issues, and to speak either English or Spanish. Once eligibility was confirmed, mothers were invited to participate in a prenatal visit in our lab or their home. Based on the results of the screening process, 209 mothers completed the prenatal visit and enrolled in the longitudinal study.

After the birth of the infant, eligibility for successive study visits was confirmed for subsequent participation. Inclusion criteria for infants included: gestational age greater than or equal to 37 weeks and no known neurological or developmental issues at birth. One mother-infant dyad was excluded from the study due to birthing complications, and three mothers unenrolled from the study. Two-hundred and five mother-infant dyads were subsequently invited to complete a lab visit when the infant was approximately 1 month of age. Of these participants, 191 mother-infant dyads completed the 1-month visit. EEG data were not collected from 27 infants either because the 1-month visit was conducted remotely (N = 21) or because the mother declined to have their infant complete the recording (N = 6). Additionally, three infants were excluded from analyses due to poor quality recordings (see below for further details), and one infant was excluded due to a technical error during EEG recording. The final sample thus included 160 mother-infant dyads. Descriptive statistics of sample demographics and study variables are presented in Table 1. The Supplementary Information presents the results of a series of independent *t*-tests and chi-square tests which compare study variables between mother-infant dyads who were either included (N = 160) or excluded (N = 31) from our analyses.

**Table 1:** Descriptive statistics of sample demographics and study variables.

Variable	Mean (standard deviation; range)	N (%)
Mother age (years)	32.31 (5.62; 19–45)	–
Gestational age at birth (weeks)	39.44 (1.05; 37.00–42.29)	–
Infant weight at birth (lbs)	7.39 (1.03; 5.00–9.94)	–
Infant age at lab visit (weeks)	5.61 (2.14; 2.14–12.86)	–
Infant sex		
Male	–	72 (45)
Female	–	88 (55)
Infant race		
White	–	63 (39)
Black or African American	–	41 (26)
Asian	–	4 (3)
American Indian/Alaska Native	–	2 (1)
Other	–	40 (25)
Refused	–	10 (6)
Infant ethnicity		
Hispanic or latino	–	73 (46)
Not hispanic or latino	–	83 (52)
Refused		4 (2)
Family Income (USD)	171,763.01 (328,494.31; 0.00–2,563,500.00)	–
Family income-to-needs	6.95 (9.47; 0.00–43.74)	–
Parental education (years)	14.98 (3.24; 6–22)	–

All mothers provided written informed consent for their family’s participation in the study. Research procedures were approved by the Institutional Review Board of Teachers College, Columbia University. All methods were performed in accordance with the Declaration of Helsinki.

### Measures

#### Prenatal visit

##### Family socioeconomic status

At the prenatal timepoint, mothers completed a questionnaire that assessed educational attainment (total years of education attained by the mother and the second parent), household composition (number of adults and children in household), and family income (estimated gross annual income). Average parental education scores were calculated by averaging the number of years of education attained by the mother and the second parent. In cases where the reporting mother was the sole parental caregiver, only maternal educational attainment was used. Maternal education scores were available for all mothers (N = 160), and second caregiver education scores were available for 153 mothers. Nine mothers selected “prefer not to answer” for the family income question; therefore, income data were available for 151 mother-infant dyads (i.e., 94% of the sample). Income-to-needs (ITN) ratios were calculated by dividing total household income by the U.S. poverty threshold for the respective family size for the year of data collection. An ITN of below 1 indicates that a family is living below the federal poverty threshold, whereas an ITN above 1 indicates that a family is living above the federal poverty threshold. Three mothers had outlying (> 3 SDs) ITN values, and so these values were winsorized prior to analyses in order to preserve power for analyses. The ITN for the sample ranged from 0 to 43.74, with a median ITN value of 2.67 (IQR = 9.17). Twenty-eight percent (N = 43) of the sample reported an ITN below the poverty line, and nine percent (N = 14) of the sample reported ITN values between 1 and 2, considered to be within the “near-poor” range. As expected, ITN values were positively skewed. Therefore, ITN values were natural log-transformed (ln) for all analyses. Additionally, ten mothers reported a family income of zero dollars. To enable log transformation, one dollar was added to all income values prior to calculating ITN. For histograms depicting the distribution of family income and ITN across the sample, see Supplementary Fig. S1.

##### Maternal physiological stress

Maternal physiological stress was indexed via hair cortisol collected at the prenatal timepoint. A small hair sample was collected from a standardized area of the posterior vertex of the mother’s scalp, with each sample weighing at least 15 mg. Each hair sample was trimmed to be approximately 3 cm long (measured from the end closest to the root), thereby containing cortisol deposited during roughly the past 3 months (based on an estimate of 1 cm hair growth per month^32^). Samples were stored at − 40 °C until being shipped for analysis. Samples were processed and analyzed using methods previously validated and described in detail^33,34^. Briefly, each sample was weighed, washed twice in isopropanol to remove external contaminants, ground to a fine powder, and extracted with methanol. The methanol extract was evaporated, redissolved in assay buffer, and analyzed in duplicate along with standards and quality controls by a sensitive and specific enzyme-linked immunosorbent assay. Assay readout was converted to pg cortisol per mg dry hair weight (pg/mg). Intra- and inter-assay coefficients of variation for this assay are < 10%. Of the 160 infants included in the final sample, 122 mothers provided hair samples. Three mothers were excluded from analyses because they were currently using steroid medications. Additionally, two mothers had outlying (> 3 SDs) hair cortisol values, and so these values were winsorized prior to analyses. Consistent with previous research^35,36^, hair cortisol values were natural log-transformed (ln) to correct for skew. In total, 119 mothers were included in hair cortisol analyses. There were no significant associations between hair cortisol and hair washing frequency, use of oral contraceptives, or use of hair dye (unadjusted *p*s > 0.71).

##### Maternal perceived stress

Maternal perceived stress was measured at the prenatal timepoint using the Perceived Stress Scale (PSS-10^37^). The PSS-10 is a 10-item self-report questionnaire that assesses the extent to which respondents perceived situations as stressful within the past month. Each item is scored on a five-point Likert scale ranging from 0 (‘Never’) to 4 (‘Very Often’). Individual items were summed to compute an overall PSS score that could range from 0 to 40, with higher scores indicating greater stress. The PSS showed good internal consistency in the current sample (α = 0.85). PSS data was available for all mothers (N = 160).

##### Maternal food insecurity

Mothers where asked if they experienced food insecurity within the past year using 3 items on the Material Deprivation Scale^38^*.* They were asked to provide a dichotomous answer (yes or no) to the following questions: Did you receive free food or meals because there wasn’t enough money? Did your child/children go hungry because there wasn’t enough money? Did you go hungry because there wasn't enough money? Individual items were summed to compute a total food insecurity score that could range from 0 to 3, with higher scores indicating greater experience of food insecurity. Food insecurity scores showed poor internal consistency in the current sample (α = 0.44). One mother selected “prefer not to answer” for all three food insecurity items, and so food insecurity data was available 159 mothers. Eighty-eight percent of the mothers (N = 140) in our sample denied experiencing food insecurity in the past year (i.e., had scores of zero). Because there was limited variability in food insecurity scores in our sample, this variable was not included in our primary analyses. See Supplementary Fig. S2 for a histogram depicting the distribution of food insecurity scores across the sample.

##### Maternal vocabulary skills

Maternal vocabulary skills were measured at the prenatal timepoint using the Picture Vocabulary Test (PVT) of the NIH Toolbox Cognition Battery^39^. The PVT assesses receptive vocabulary via auditory comprehension of single words that are graded in difficulty using an auditory word-picture matching paradigm. On each trial, mothers were shown four high-resolution color photos on an iPad while simultaneously hearing a word. They were then instructed to select the picture that best matches the word they heard. The PVT included two practice items followed by 25 test items, the difficulty of which depended on the mother’ initial performance. Mothers’ performance on the PVT was converted to a theta score (ranging from 4 to − 4), based on item response theory. PVT data for one mother was not collected due to a technical error. Therefore, PVT data was available for 159 mothers.

#### One-month visit

##### Feeding style

At the 1-month time point, mothers were asked how they were currently feeding their infant (i.e., What do you currently feed your baby?). Response options included, “Only breast milk”, “Only baby formula”, and “Both breast milk and baby formula”. One mother selected “prefer not to answer” to the feeding style item, and so feeding style data was available 159 mothers. Sixty-seven mothers (42%) reported exclusively breastfeeding their infant, 68 mothers (43%) reported feeding their infant both breast milk and formula, and 24 (15%) mothers reported exclusively feeding their infant formula.

##### Infant demographic and health-related factors

Infants’ gestational age at birth (in weeks), weight at birth (in lbs), assigned sex, race, ethnicity, and age at the 1-month lab visit were collected from mothers via questionnaire at the 1-month time point. Ten mothers selected “prefer not answer” on the item that assessed infants’ race, and four mothers selected “prefer not answer” on the item that assessed infants’ ethnicity. Therefore, infant race and ethnicity data were available 150 and 156 infants, respectively.

### Electroencephalography data acquisition and processing

EEG was recorded using a 128-channel montage HydroCel Geodesic Sensor Net (Magstim Electrical Geodesic Inc., Eugene, OR, USA) and on a Net Amps 400 amplifier. Whenever possible, all electrode impedances were kept below 50 kΩ. The sampling rate was 1000 Hz and data were online referenced to the vertex (Cz) electrode. EEG recordings were completed in a dimly lit, sound-attenuated room with low electrical signal background. Infants were held by their mothers as they viewed a wordless (but not soundless) video^40^. Infants were positioned approximately 60 cm from a Lenovo ThinkVision monitor (model T2054pC) held backward over their mother’s shoulder or seated and facing forward on her lap. The auditory stimuli in the video facilitated infants’ engagement with video, ensuring that they remained attentive during the recording. Mothers were instructed to talk and interact with their infant as little as possible. An experimenter stood in the recording room with the mother and infant, and if needed, used a quiet toy to redirect the infant’s attention to the screen. Data collection lasted approximately five minutes (M = 5.34, *SD* = 0.84) unless the infant rejected collection before that time due to fussiness/fatigue. Data from additional tasks and questionnaires not discussed here are reported elsewhere^41,42^.

Offline processing was completed using the EEGLAB toolbox (version 2022.1^43^) MATLAB (Version R2021b; The MathWorks, Natick, MA), and the MADE pipeline^44^. Prior to filtering, the outermost ring of electrodes (i.e., electrodes located near the base of the skull: 17, 38, 43, 44, 48, 49, 113, 114, 119, 120, 121, 125, 126, 127, 128, 56, 63, 68, 73, 81, 88, 94, 99, 107) were removed because these electrodes tend to have poor connections and are highly susceptible to noise in infant samples^44^. Data were then high-pass filtered at 0.30 Hz and low-pass filtered at 50 Hz. Bad channels were identified and removed using the EEGLAB plug-in FASTER^45^. Ocular artifacts and generic noise were removed by creating a copy of the dataset and performing independent component analysis (ICA) on the copied data. The copied dataset was high-pass filtered at 1 Hz and segmented into arbitrary 1000 ms epochs. Epochs were removed from this copied dataset if the amplitude was ± 1000 μV or if power in the 20–40 Hz band (after Fourier analysis) was greater than 30 dB^23,35^. If more than 20% of the epochs in a given channel were removed, that channel was excluded from both the ICA-copied dataset and the original dataset^35,46^. Additionally, if an infant had all of their channels deemed globally bad across all epochs by FASTER, that infant was excluded from subsequent processing (removed N = 2).

Following this, ICA^47,48^ was performed on the copied dataset and the ICA weights were copied back to the original continuous data (high-pass filtered at 0.30 Hz). The adjusted-ADJUST toolbox^49^ was used to automatically identify artifactual independent components (ICs) in the original dataset. The first 35 ICs were also visually inspected to identify any remaining artifactual ICs. All artifactual ICs were removed from the data. Next, data were epoched in segments of one second with 50% (500 ms) overlap. Consistent with prior work^46^, epochs where any of the frontal electrodes (i.e., 1, 8, 14, 21, 25, 32) exceeded a voltage threshold of ± 150 μV were rejected from all further analyses to ensure ocular artifacts were removed. For the remaining epochs, bad channels were identified if the electrode exceeded a voltage threshold of ± 150 μV. Epochs were rejected when more than 10% of channels were interpolated^23,35^. If all epochs were deemed artifactual for an infant (i.e., more than 10% of channels were interpolated on every epoch), that infant was excluded from subsequent processing (removed N = 1). All rejected channels were interpolated. Finally, data were re-referenced to an average reference.

A Fast Fourier Transform (FFT) with a Hamming Window was applied to the epoched data. Consistent with other infant studies^22,23,35^, spectral power (μV^2^) across all epochs was computed for theta (3–5 Hz), alpha (6–9 Hz), beta (13–19 Hz), and gamma frequency ranges (21–45 Hz). Log_10_-transformed absolute and relative power was calculated for each of the four frequency bands. These values were then averaged across all included electrodes to create estimates of whole-brain absolute and relative theta, alpha, beta, and gamma power. Whole-brain relative power was calculated by dividing absolute power within each frequency band (e.g., whole-brain theta) by total absolute power from all frequency bands (i.e., whole-brain theta, alpha, beta, and gamma). There were outliers (> 3 SDs) in whole-brain relative alpha (n = 1), beta (n = 2), and gamma power (n = 3) as well as in whole-brain absolute alpha (n = 1), beta (n = 2), and gamma power (n = 4). Excluding infants with outlying relative and absolute power values from our analyses did not change the pattern of results we observed. Therefore, infants with outlying relative and absolute power values were included in our analyses.

In addition to computing whole-brain activity, we also selected five candidate regions of interest (ROI) to score each of the four frequency bands based on prior research that has examined regional EEG power in infant samples^22^. Specifically, we calculated absolute and relative regional power by averaging electrodes within the following five ROIs (see Supplementary Fig. S3): frontal (20, 12, 5, 118, 24, 19, 11, 4, 124, 27, 23, 18, 16, 10, 3, 123), central (6, 112, 105, 87, 79, 54, 37, 30, 13, 106, 80, 55, 31, 7, Cz), parietal (53, 61, 62, 78, 86, 52, 60, 67, 72, 77, 85, 92), temporal (40, 46, 39, 45, 50, 109, 102, 115, 108, 101), and occipital (66, 71, 76, 84, 70, 75, 83). Regional relative power was calculated by averaging absolute power across the candidate electrodes in a region (e.g., frontal) and then dividing the power within each frequency band (e.g., theta) by total absolute power from all frequency bands. This was performed in each of the five ROIs. Given that relative power minimizes individual differences in absolute power resulting from variations in age at assessment, skull thickness and other anatomic factors^13^, and because prior research has demonstrated associations between deprivation and relative power^20,26,50^, relative power was used in the analyses that follow. Absolute power results are presented in the Supplementary Information.

To determine the minimum number of epochs needed to obtain reliable estimates of theta, alpha, beta, and gamma power, we used the Spearman-Brown split-half correlation procedure with multiple iterations (for more details about this procedure, see^40,49^). The minimum number of epochs needed to achieve an acceptable reliability cut-off (i.e., α = 0.70) for each frequency band were as follows: 5 epochs for beta and gamma power, 7 epochs for theta power, and 15 epochs for alpha power. All infants in our sample (N = 160) had at least 26 artifact-free epochs (M = 418.04, *SD* = 153.66, range = 26–832) and were therefore included in subsequent analyses. The number of artifact-free epochs was not significantly associated with prenatal family ITN (*r* = 0.11, unadjusted *p* = 0.17), parental education (*r* = 0.13, unadjusted *p* = 0.10) or infant age (in weeks) at the 1-month lab visit (*r* = 0.02, unadjusted *p* = 0.88).

### Statistical analyses

All statistical analyses were conducted in SPSS (Version 28) or in R software (Version 4.2.3). For descriptive purposes, we calculated Pearson’s *r* to examine bivariate associations among study variables of interest.

Next, to examine whether prenatal family ITN and parental education were associated with differences in infant brain activity, we conducted eight simultaneous regressions (i.e., four regressions with ITN as a predictor, and four regressions with parental education as a predictor). In each of these analyses, whole-brain relative power (i.e., either theta, alpha, beta, or gamma) was the outcome measure, and family ITN or parental education was entered as the predictor. Infant age at the 1-month lab visit (in weeks), sex (0 = male, 1 = female), cohort number (0 = Cohort 1, 1 = Cohort 2), and number of EEG epochs retained were included as covariates.

Following this, and to determine whether significant associations between prenatal family ITN, parental education and infant brain activity were stronger over certain regions of the scalp (i.e., frontal, central, parietal, temporal, and occipital), we conducted multiple follow-up simultaneous regressions. In each of these regressions, regional relative power was the outcome measure, and family ITN or parental education was entered as the predictor. Infant age at the 1-month lab visit, sex, cohort number, and number of EEG epochs retained were included as covariates.

### Sensitivity analyses

To examine the potentially confounding influence of other prenatal and postnatal experiences, as well as other infant demographic and health-related factors, we conducted sensitivity analyses that included additional model covariates. Specifically, we repeated our primary simultaneous regression analyses with whole-brain relative EEG power, but also included the following additional covariates: maternal prenatal physiological stress (i.e., hair cortisol), maternal prenatal perceived stress, maternal prenatal receptive vocabulary, postpartum exclusive breastfeeding (1 = exclusively breast milk fed, 0 = exclusively formula fed, 0 = mixed breast milk and formula fed), postpartum exclusive formula feeding (1 = exclusively formula fed, 0 = exclusively breast milk fed, 0 = mixed breast milk and formula fed), infant race (0 = White, 1 = Non-white), infant ethnicity (0 = Non-Hispanic/Latino, 1 = Hispanic/Latino), infant weight at birth (in lbs), and infant gestational age at birth (in weeks). In these models, we also included both prenatal family ITN and parental education to examine unique associations between different measures of SES and infant EEG power. As expected, prenatal family ITN and parental education were significantly associated, but they did not violate assumptions of multicollinearity (VIF < 2.5).

To account for multiple comparisons, false discovery rate (FDR) corrections were applied to all analyses using the *p.adjust* package in R.

The study’s deidentified data as well as syntax used for all analyses are available at the following link: https://osf.io/w9evp/. The code and stimuli for the resting EEG task are available from the corresponding author upon request.

## Results

Table 2 presents descriptive statistics and bivariate associations among study variables (results for absolute power are presented in Supplementary Table S1).

**Table 2:** Descriptive statistics and associations among study variables.

	1	2	3	4	5	6	7	8	9	10	11	12	13	14	15	16	17	18
1. Family income-to-needs (ln)	–																	
2. Parental education (years)	0.53**	–																
3. Maternal hair cortisol (ln; pg/mg)	− 0.18	− 0.12	–															
4. Maternal perceived Stress	− 0.19*	0.00	0.08	–														
5. Food insecurity	− 0.14	− 0.17*	0.05	0.34**	–													
6. Maternal receptive vocabulary	0.40**	0.59**	0.05	− 0.07	− 0.16*	–												
7. Breastfed	0.33**	0.42**	− 0.07	− 0.02	− 0.14	0.37**	–											
8. Formula fed	− 0.26**	− 0.31**	0.00	0.02	0.10	− 0.21**	− 0.36**	–										
9. Infant gestational age at birth (weeks)	0.13	0.09	− 0.08	− 0.06	− 0.05	0.13	0.21**	− 0.13	–									
10. Infant weight at birth (lbs)	0.00	− 0.01	0.02	− 0.07	− 0.22**	0.01	0.15	− 0.11	0.31**	–								
11. Infant sex	0.06	0.05	0.03	− 0.07	0.04	0.09	0.01	− 0.04	0.09	− 0.18*	–							
12. Infant race	− 0.30**	− 0.50**	0.14	0.04	0.05	− 0.41**	− 0.20*	0.11	− 0.06	0.02	− 0.02	–						
13. Infant ethnicity	− 0.39**	− 0.47**	0.10	− 0.12	0.18*	− 0.34**	− 0.31**	0.29**	− 0.04	− 0.14	0.09	0.26**	–					
14. Infant age at lab visit (weeks)	0.01	0.00	0.25**	− 0.01	0.03	0.06	0.07	− 0.20*	− 0.22**	− 0.24**	0.01	0.02	0.01	–				
15. Infant relative theta (log_10_; μV^2^)	− 0.16	− 0.05	0.02	0.15	0.06	0.03	− 0.16*	0.06	− 0.15	0.06	− 0.05	− 0.10	0.11	0.05	–			
16. Infant relative alpha (log_10_; μV^2^)	0.02	− 0.07	− 0.11	− 0.16*	− 0.01	− 0.15	0.04	0.07	0.14	− 0.03	0.11	0.22**	− 0.01	− 0.23**	− 0.66**	–		
17. Infant relative beta (log_10_; μV^2^)	0.17*	0.11	− 0.01	− 0.08	− 0.03	0.04	0.10	− 0.04	0.12	− 0.04	0.02	0.00	− 0.12	− 0.06	− 0.83**	0.28**	–	
18. Infant relative gamma (log_10_; μV^2^)	0.17*	0.13	0.12	− 0.04	− 0.08	0.12	0.21**	− 0.18*	0.03	− 0.05	− 0.05	− 0.09	− 0.13	0.25**	− 0.57**	− 0.19*	0.61**	–
Mean	0.30	14.98	2.04	13.10	0.14	4.26	–	–	39.44	7.39	–	–	–	5.61	0.69	0.23	0.06	0.03
SD	3.27	3.24	1.10	7.06	0.43	2.85	–	–	1.05	1.03	–	–	–	2.14	0.04	0.02	0.01	0.02
N	151	160	119	160	159	159	159	159	160	160	160	150	156	160	160	160	160	160

Breastfed (1 = exclusively breastmilk fed, 0 = exclusively formula fed, 0 = mixed breastmilk and formula fed); Formula fed (1 = exclusively formula fed, 0 = mixed breastmilk and formula fed, 0 = exclusively breastmilk fed); Infant Sex (0 = Male, 1 = Female); Infant Race (0 = White, 1 = Non-white); Infant Ethnicity (0 = Non-Hispanic/Latino, 1 = Hispanic/Latino). Unadjusted **p < 0.01 *p < 0.05.

### Association between prenatal family ITN and infant whole-brain relative EEG power

We first examined whether prenatal family ITN was associated with whole-brain relative EEG power when controlling for infant age at the 1-month lab visit, sex, number of epochs, and cohort number (see Table 3; results for absolute power are presented in Supplementary Table S2). Results indicated that higher prenatal family ITN was significantly associated with less whole-brain relative theta power (*β* = − 0.18, FDR-adjusted *p* = 0.03, 95% CI [− 0.34, − 0.03]). Higher prenatal family ITN was also associated with more whole-brain relative beta power (*β* = 0.20, FDR-adjusted *p* = 0.03, 95% CI [0.04, 0.35]) and more whole-brain relative gamma power (*β* = 0.17, FDR-adjusted *p* = 0.03, 95% CI [0.02, 0.32]) when controlling for covariates. However, prenatal family ITN was not significantly associated with whole-brain relative alpha power (*β* = 0.05, FDR-adjusted *p* = 0.51, 95% CI [− 0.10, 0.20]).

**Table 3:** Results of the simultaneous multiple regressions examining associations between prenatal family income-to-needs (Model 1) and parental education (Model 2) with whole-brain relative theta, alpha, beta, and gamma power in infants.

Predictors	Relative theta		Relative alpha		Relative beta		Relative gamma	
	β	95%	β	95% CI	β	95% CI	β	95% CI
Model 1 (N = 151)								
Family income-to-needs (ln)	− 0.18*	− 0.34, − 0.03	0.05	− 0.10, 0.20	0.20*	0.04, 0.35	0.17*	0.02, 0.32
Infant age (weeks)	0.08	− 0.08, 0.24	− 0.24**	− 0.40, − 0.08	− 0.10	− 0.26, 0.07	0.23**	0.08, 0.39
Infant sex	− 0.10	− 0.26, 0.05	0.11	− 0.04, 0.26	0.08	− 0.07, 0.24	0.00	− 0.16, 0.15
Number of epochs	0.39**	0.22, 0.56	− 0.20*	− 0.36, − 0.03	− 0.34**	− 0.51, − 0.17	− 0.29**	− 0.46, − 0.12
Cohort	− 0.10	− 0.27, 0.07	− 0.10	− 0.27, 0.06	0.11	− 0.06, 0.28	0.26**	0.10, 0.43
	R^2^ = 0.15, *F*(5, 145) = 5.21**		R^2^ = 0.14, *F*(5, 145) = 4.60**		R^2^ = 0.13, *F*(5, 145) = 4.31**		R^2^ = 0.19, *F*(5, 145) = 6.63**	
Model 2 (N = 160)								
Parental education (years)	− 0.08	− 0.23, 0.07	− 0.04	− 0.19, 0.12	0.13	− 0.02, 0.28	0.12	− 0.03, 0.27
Infant age (weeks)	0.06	− 0.10, 0.21	− 0.20*	− 0.36, − 0.05	− 0.07	− 0.22, 0.09	0.21*	0.07, 0.36
Infant sex	− 0.09	− 0.25, 0.06	0.12	− 0.03, 0.27	0.06	− 0.10, 0.21	− 0.01	− 0.16, 0.13
Number of epochs	0.32**	0.15, 0.48	− 0.14	− 0.31, 0.02	− 0.30**	− 0.47, − 0.14	− 0.25**	− 0.41, − 0.09
Cohort	− 0.06	− 0.22, 0.11	− 0.13	− 0.29, 0.04	0.07	− 0.10, 0.24	0.24*	0.07, 0.40
	R^2^ = 0.10, *F*(5, 154) = 3.29**		R^2^ = 0.12, *F*(5, 154) = 4.03**		R^2^ = 0.09, *F*(5, 154) = 3.17**		R^2^ = 0.15, *F*(5, 154) = 5.59**	

Infant Sex (0 = Male, 1 = Female); Cohort (0 = Cohort 1, 1 = Cohort 2); FDR-adjusted **p < 0.01 *p < 0.05.

**Figure 1:**
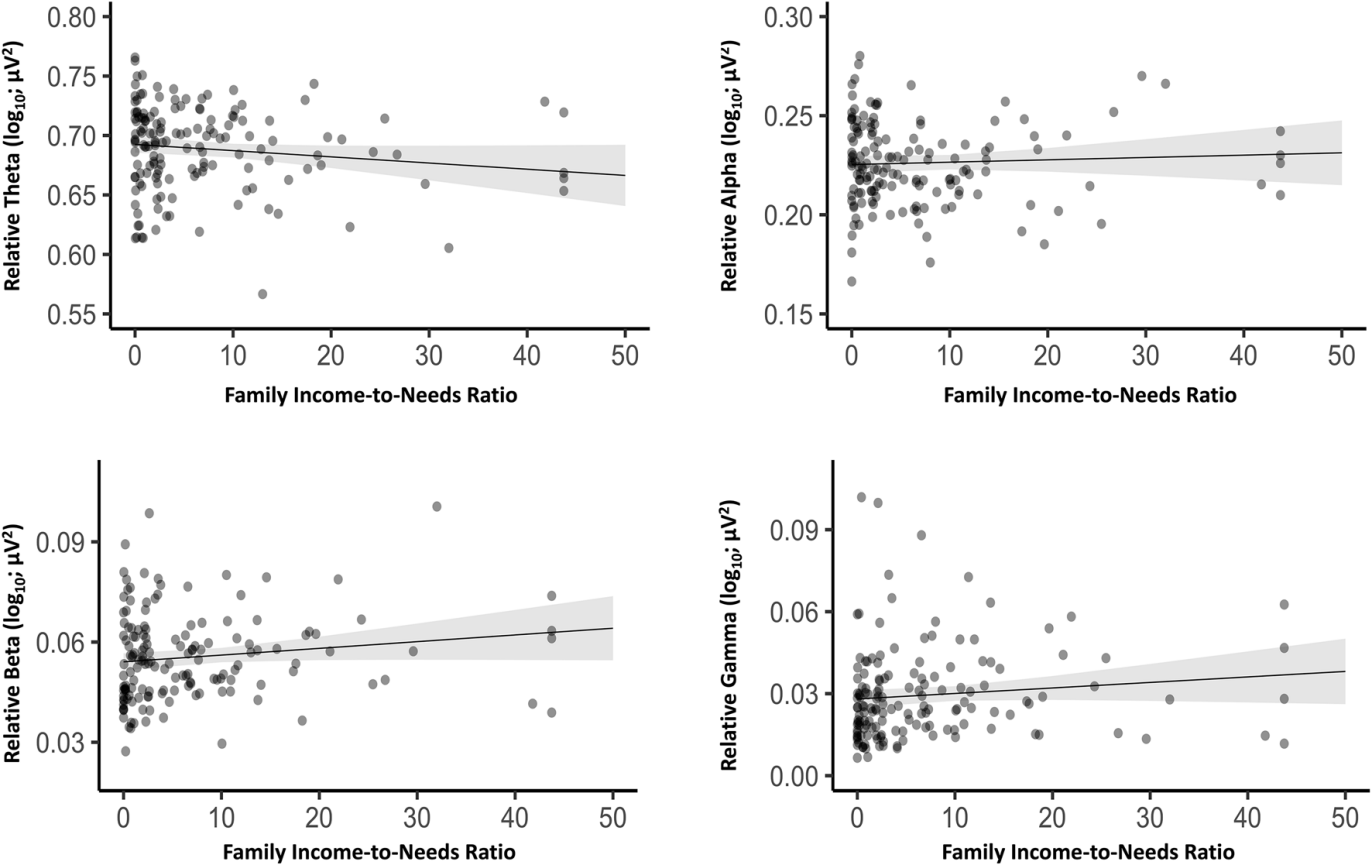
Partial regression plots depicting associations between prenatal family income-to-needs and relative theta (β = − 0.18, FDR-adjusted *p* = 0.03), alpha (β = 0.05, FDR-adjusted *p* = 0.51), beta (β = 0.20, FDR-adjusted *p* = 0.03), and gamma (β = 0.17, FDR-adjusted *p* = 0.03) power in infants, adjusting for the effects of infant age, sex, number of epochs, and cohort number (N = 151). Note. To better illustrate and interpret associations between prenatal family ITN and relative whole-brain power, the plots do not use log-transformed family ITN. However, log-transformed family ITN was used in our regression analyses. A family ITN of 1 means that the family’s annual income placed them at the federal poverty threshold. Shaded areas represent 95% confidence intervals.

**Figure 2:**
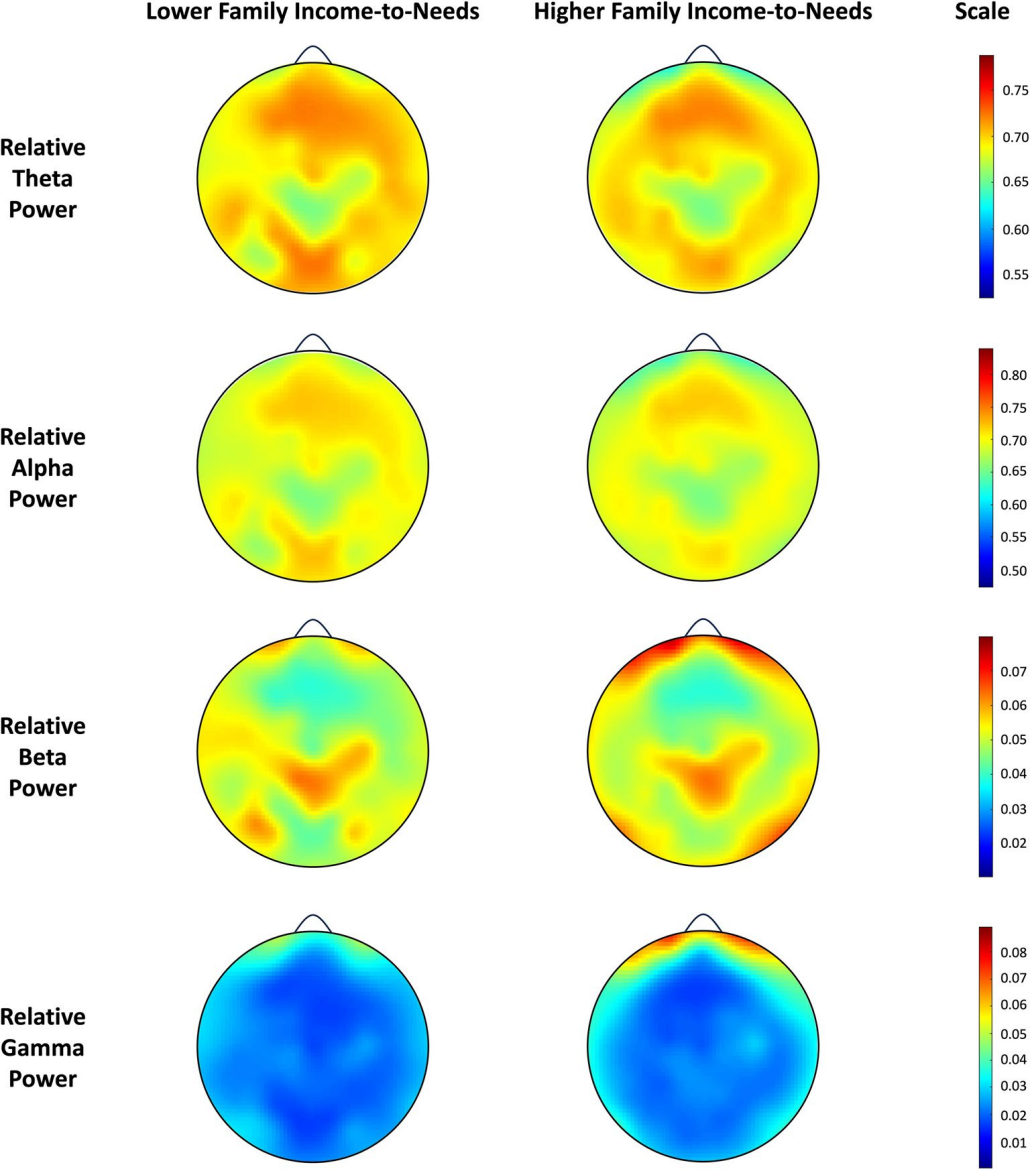
Topographic heat maps of the distribution of relative theta, alpha, beta, and gamma power across the scalp for infants. Data are presented using a median split for visualization purposes only. Families with lower prenatal family income-to-needs ratios are presented in the left column and families with higher prenatal family income-to-needs ratios are presented on the right. Note. Warmer colors represent more power in each respective frequency band.

Figure 1 presents partial regression plots depicting associations between prenatal family ITN and whole-brain relative theta, alpha, beta, and gamma power, adjusting for the effects of infant age at the 1-month lab visit, sex, number of epochs and cohort number. Figure 2 shows topographic heat maps of the distribution of EEG relative power across the scalp within each of the four frequency bands. For presentation purposes, a median split of family ITN was used to depict infants from families with lower prenatal ITN ratios (i.e., below the median) vs. infants from families with higher prenatal ITN ratios (i.e., above the median).

We repeated our analyses examining the associations between prenatal family ITN and whole-brain relative EEG power excluding infants older than 2 months of age (N = 14), excluding families with ITN ratios greater than 20 (N = 12), and when not log-transforming ITN (see Supplementary Tables S3, S4, and S5, respectively). The results of these analyses indicated similar effect sizes between prenatal family ITN and relative theta, beta, and gamma power in infants, with smaller *p*-values (*p*s ranged from 0.06 to 0.19).

### Association between prenatal family ITN and infant regional relative EEG power

Follow-up regional analyses indicated that higher prenatal family ITN was associated with less relative theta power (*β* = − 0.17, FDR-adjusted *p* = 0.04, 95% CI [− 0.33, − 0.02]), as well as more relative beta power (*β* = 0.17, FDR-adjusted *p* = 0.04, 95% CI [0.02, 0.33]) and more relative gamma power (*β* = 0.16, FDR-adjusted *p* = 0.05, 95% CI [0.01, 0.31]) in occipital regions. Higher prenatal family ITN was also associated with more relative gamma power in the parietal region (*β* = 0.19, FDR-adjusted *p* = 0.05, 95% CI [0.03, 0.35]). Associations between prenatal family ITN and EEG power at other regions were not significant (FDR-adjusted *ps* > 0.11).

### No association between prenatal parental education and infant whole-brain relative EEG power

We next examined whether prenatal parental education was associated with whole-brain relative EEG power when controlling for infant age at the 1-month lab visit, sex, number of epochs, and cohort number (See Table 3; results for absolute power are presented in Supplementary Table S2). Results indicated that parental education was not significantly associated with whole-brain relative theta, alpha, beta, or gamma power when controlling for covariates.

We repeated our analyses examining the associations between prenatal family ITN, parental education and whole-brain relative and absolute EEG power excluding the infants with outlying relative and absolute EEG power values (see Supplementary Table S6). The results of these analyses were very similar to the results reported here.

### Sensitivity analyses

Finally, we performed sensitivity analyses to test the robustness of our findings by examining whether associations between prenatal family ITN and infant whole-brain relative EEG power held when adjusting for the influence of other measured prenatal and postnatal experiences; infant demographic and health-related factors; and parental education (See Table 4; for results excluding infants with outlying relative power values see Supplementary Table S7). We again found that higher prenatal family ITN was significantly associated with less whole-brain relative theta power (*β* = − 0.24, FDR-adjusted *p* = 0.04, 95% CI [− 0.44, − 0.04]), as well as more whole-brain relative beta power (*β* = 0.23, FDR-adjusted *p* = 0.04, 95% CI [0.02, 0.45]) and more whole-brain relative gamma power (*β* = 0.24, FDR-adjusted *p* = 0.04, 95% CI [0.02, 0.46]) when controlling for covariates. Prenatal family ITN was not significantly associated with whole-brain relative alpha power (*β* = 0.06, FDR-adjusted *p* = 0.54, 95% CI [− 0.13, 0.25]) when controlling for covariates. Moreover, parental education was not significantly associated with whole-brain relative theta, alpha, beta, or gamma power when controlling for covariates (*p*s > 0.88), indicating that socioeconomic-related differences in infant EEG power were specific to family ITN and not parental education.

**Table 4:** Sensitivity analyses examining the association between prenatal family income-to-needs and infant relative EEG power when adjusting for the effects of parental education, maternal prenatal hair cortisol, maternal prenatal perceived stress, maternal prenatal receptive vocabulary, postpartum feeding style, infant gestational age at birth, infant weight at birth, infant sex, infant race, infant ethnicity, infant age at the one-month lab visit, number of usable epochs, and cohort number.

N = 103	Relative theta		Relative alpha		Relative beta		Relative gamma	
Predictors	β	95% CI	β	95% CI	β	95% CI	β	95% CI
Family income-to-needs (ln)	− 0.24*	− 0.44, − 0.04	0.06	− 0.13, 0.25	0.23*	0.02, 0.45	0.24*	0.02, 0.46
Parental education (years)	0.02	− 0.23, 0.27	− 0.12	− 0.36, 0.12	0.10	− 0.18, 0.37	0.04	− 0.23, 0.32
Maternal hair cortisol (ln; pg/mg)	− 0.10	− 0.29, 0.10	0.02	− 0.16, 0.21	0.14	− 0.08, 0.36	0.06	− 0.15, 0.28
Maternal perceived stress	0.08	− 0.10, 0.27	− 0.15	− 0.33, 0.03	0.01	− 0.19, 0.22	0.02	− 0.18, 0.23
Maternal receptive vocabulary	0.09	− 0.14, 0.31	− 0.05	− 0.27, 0.17	− 0.04	− 0.29, 0.21	− 0.08	− 0.33, 0.17
Breastfed	− 0.10	− 0.29, 0.10	0.15	− 0.04, 0.33	0.02	− 0.20, 0.23	− 0.01	− 0.22, 0.20
Formula fed	0.05	− 0.18, 0.28	− 0.01	− 0.22, 0.21	− 0.03	− 0.28, 0.22	− 0.07	− 0.32, 0.17
Infant gestational age at birth (weeks)	− 0.27*	− 0.46, − 0.08	0.10	− 0.08, 0.28	0.22	0.01, 0.42	0.28*	0.07, 0.48
Infant weight at birth (lbs)	0.20	0.00, 0.40	− 0.09	− 0.28, 0.10	− 0.19	− 0.41, 0.03	− 0.16	− 0.37, 0.06
Infant sex	0.02	− 0.16, 0.20	0.06	− 0.11, 0.23	− 0.07	− 0.26, 0.13	− 0.07	− 0.26, 0.13
Infant race	− 0.08	− 0.27, 0.12	0.18	0.00, 0.36	0.03	− 0.18, 0.24	− 0.11	− 0.32, 0.10
Infant ethnicity	0.15	− 0.09, 0.39	− 0.17	− 0.40, 0.06	− 0.11	− 0.38, 0.15	0.00	− 0.26, 0.26
Infant age at lab visit (weeks)	0.03	− 0.15, 0.21	− 0.21*	− 0.38, − 0.04	− 0.05	− 0.25, 0.14	0.27*	0.07, 0.46
Number of epochs	0.34**	0.16, 0.52	− 0.14	− 0.32, 0.03	− 0.27*	− 0.47, − 0.07	− 0.31**	− 0.51, − 0.12
Cohort	0.03	− 0.20, 0.25	− 0.17	− 0.39, 0.05	− 0.10	− 0.35, 0.15	0.25	0.00, 0.50
	R^2^ = 0.33, *F*(15, 87) = 2.89**		R^2^ = 0.32, *F*(15, 87) = 2.77**		R^2^ = 0.25, *F*(15, 87) = 1.91*		R^2^ = 0.33, *F*(15, 87) = 2.82**	

Breastfed (1 = exclusively breastmilk fed, 0 = exclusively formula fed, 0 = mixed breastmilk and formula fed); Formula fed (1 = exclusively formula fed, 0 = mixed breastmilk and formula fed, 0 = exclusively breastmilk fed); Infant Sex (0 = Male, 1 = Female); Infant Race (0 = White, 1 = Non-white); Infant Ethnicity (0 = Non-Hispanic/Latino, 1 = Hispanic/Latino); Cohort (0 = Cohort 1, 1 = Cohort 2); FDR-adjusted **p < 0.01 *p < 0.05.

See the Supplementary Information for the results of an additional robustness check comparing the strength of associations between prenatal family ITN, parental education, and relative EEG power.

## Discussion

Here we examined whether prenatal family SES is associated with patterns of resting brain activity in 1-month old infants. Consistent with our hypotheses, higher prenatal family income-to-needs was associated with less lower-frequency (theta) power and more higher-frequency (beta and gamma) power in infants. These associations were specific to family income, and not parental education, and were most apparent over occipital and parietal regions of the brain. Moreover, associations between prenatal family income-to-needs and infant brain activity held after adjusting for various prenatal and postnatal experiences, as well as infant demographic and health factors.

These results are consistent with prior work demonstrating associations between family SES and individual differences in lower- and higher frequency power in young children^20,22,23^, and extends this work by showing that prenatal income-related differences in brain activity are detectable within the first month of life. Resting EEG power in infants is moderately heritable^51^, but is also subject to substantial environmental influence^15,35^. The variation in brain activity observed here might thus be due to genetic factors and/or due to more proximal, prenatal or early postnatal experiences that vary with SES. In our study, however, associations between prenatal family income-to-needs and infant brain activity were independent of a variety of measured prenatal experiences and characteristics (maternal perceived and physiological stress, maternal vocabulary), postnatal experiences (feeding style), as well as infant characteristics (demographic factors, gestational age, birth weight). Nonetheless, since our study did not directly assess genetic factors, nor did it measure other important prenatal (e.g., maternal prenatal nutrition, exercise, sleep quality, toxin exposure) and postnatal experiences (e.g., stress, parenting), we are unable to rule out these potential influences on infant brain function.

Our findings align with prior research suggesting that family socioeconomic disadvantage may contribute to adaptations in the normative development of brain activity in infants^7,8^. The patterns of brain activity we observed in infants from lower income families have been linked to lower scores on subsequent language^18^, cognitive^29^ and socio-emotional outcomes^16^, as well as with the development of psychopathology^19^, suggesting that income-related differences in brain activity may be early markers for risk of future developmental and health outcomes. We note, however, that children’s brain development reflects an adaptation to their lived experiences^52^, and that not all children living in families facing socioeconomic disadvantage will show alterations in their brain activity. Indeed, the patterns of brain activity observed here may support the development of skills that are advantageous in the context of deprivation^53^. Thus, adaptation does not necessarily reflect dysfunction or deficit, but rather an expected and appropriate response to the environment^54^.

Interestingly, prenatal family income, but not parental education, was associated with infant brain function. These data are consistent with the results of some previous studies^22,24,30^, and underscore the fact that family income and parental education reflect distinct aspects of family SES which capture meaningful differences in children’s early experiences^31^. It is possible that income-related experiences (e.g., nutrition, access to medical care, exposure to toxins, stress) might more strongly impact brain function very early in childhood (e.g.,^28^), whereas parental education may more directly influence the parenting environment, which may subsequently affect brain function later in childhood.

Our follow-up exploratory analyses indicated some regional specificity. For instance, higher prenatal family income-to-needs was associated with less lower-frequency (theta) power in occipital regions, as well as more higher-frequency power in occipital (beta and gamma) and parietal (gamma) regions, consistent with prior research^23,50,55^. Family SES-related differences in regional brain activity have also been observed in other regions (e.g., frontal, central and temporal) in young children^20–23,28^, suggesting that family SES may have widespread associations with regional EEG power, or that regional associations change with development. Given that brain maturation in early childhood may progress from back-to-front^56^, environmental influences might be particularly pronounced in occipital regions during early infancy.

This study has several strengths, including its simultaneous examination of the associations among prenatal family income-to-needs, parental education, and resting brain activity in a relatively large, socioeconomically, and racially/ethnically diverse sample of very young infants. However, several limitations should be considered. First, our cross-sectional and correlational study design precludes causal inferences, and it remains unclear whether observed differences in infant brain activity represent temporary or stable developmental differences. Second, the effect sizes observed in the current study were small in magnitude, highlighting that prenatal family income is likely one of many factors that may shape infant brain activity. Third, the number of usable EEG epochs in our sample was a significant predictor of theta, alpha, beta and gamma power. The reasons for these associations are unclear but might be due to individual differences in infants’ state-related arousal, cognition or affect during EEG recordings^57^. Without behavioral data gathered during EEG collection, contributions of infant state to the EEG patterns observed are unknown. Finally, we used a traditional approach to classifying predefined canonical frequency bands in infants. However, the most appropriate ranges to define such bands in 1-month old infants are not firmly established. Future studies may consider employing spectral parameterization approaches, which are agnostic to predefined canonical frequency bands^58^.

In conclusion, the present study suggests that prenatal family income-to-needs is associated with developmental differences in infant brain activity within the first month of life. These associations are specific to family income, and not parental education, and are independent of a variety of measured prenatal and postnatal experiences, as well as infant demographic and health-related factors. Structural inequities associated with socioeconomic disadvantage—such as financial hardship, housing and food insecurity, unemployment, lack of access to childcare, racism, and discrimination—may exert a particularly pronounced impact on family income, potentially driving the findings reported here. Although preliminary and in need of replication, our findings suggest that investment in income-support programs and policies during pregnancy may hold promise for supporting children’s early brain development.

### Supplementary Information

Supplementary Information.

## Data Availability

The study’s deidentified data as well as syntax used for all analyses are available at the following link: https://osf.io/w9evp/. The code and stimuli for the resting EEG task are available from the corresponding author upon request.
