# Supplementary material for: Prenatal family income, but not parental education, is associated with resting brain activity in 1-month-old infants
Source: Sci Rep. 2024 Jun 13;14:13638. doi: 10.1038/s41598-024-64498-3 (PMC11176315; doi:10.1038/s41598-024-64498-3)
Supplement: Supplementary file 1 — Supplementary Information. [file 41598_2024_64498_MOESM1_ESM.pdf]

Lower prenatal family income, but not parental education, is associated with resting brain activity in one-month old infants

Supplementary Information

Comparisons between families who did or did not contribute infant EEG data

Of the 191 mother-infant dyads who completed the one-month visit, EEG was not collected from 31 infants. We calculated a series of independent *t*-tests and chi-square tests to examine whether families who were included (N = 160) or excluded (N = 31) from our analyses differed on study variables. Mothers who were included in our analyses did not differ in age ($t(189) = 0.63$, $p = .53$), race ($\chi(5) = 3.47$, $p = .63$), ethnicity ($\chi(1) = 0.12$, $p = .73$), perceived stress ($t(189) = 1.47$, $p = .15$), physiological stress ($t(142) = 0.22$, $p = .83$), receptive vocabulary ($t(186) = 0.08$, $p = .94$), family ITN ($t(176) = 0.99$, $p = .32$), parental education ($t(189) = 1.07$, $p =.29$), exclusive breastfeeding ($\chi(1) = 0.00$, $p = .98$) or exclusive formula feeding ($\chi(1) = 1.13$, $p = .15$) from mothers who were excluded from our analyses. Additionally, infants who were included in our analyses did not differ in assigned sex ($\chi(1) = 0.10$, $p = .75$), race ($\chi(5) = 9.03$, $p = .11$), ethnicity ($\chi(2) = 0.17$, $p = .92$), weight at birth ($t(189) = 0.56$, $p = .57$) or gestational age at birth ($t(189) = 1.72$, $p = .09$). However, infants who were included in our analyses (M = 5.61, *SD* = 2.14) were significantly younger at the one-month lab visit than infants who were excluded (M = 9.45, *SD* = 5.29; $t(189) = 6.81$, $p < .001$).

Figure S1

Histograms depicting the distribution of family income (x-axis, bin width of 10,000) and income-to-needs (x-axis, bin width of 1) across the sample (y-axis, n = 160).

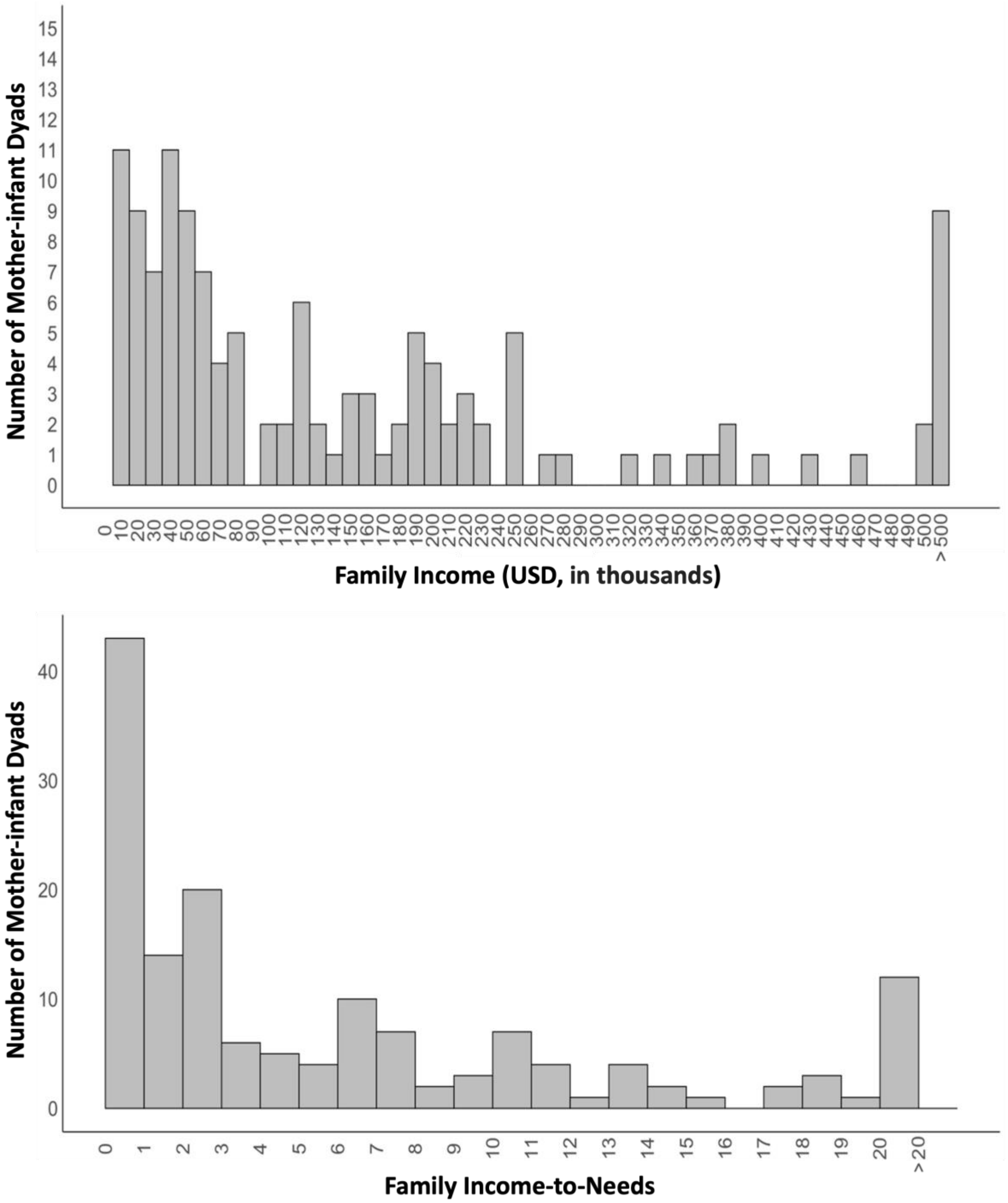

Figure S2

Histogram depicting the distribution of prenatal food insecurity scores (x-axis) across the sample (y-axis, n = 159).

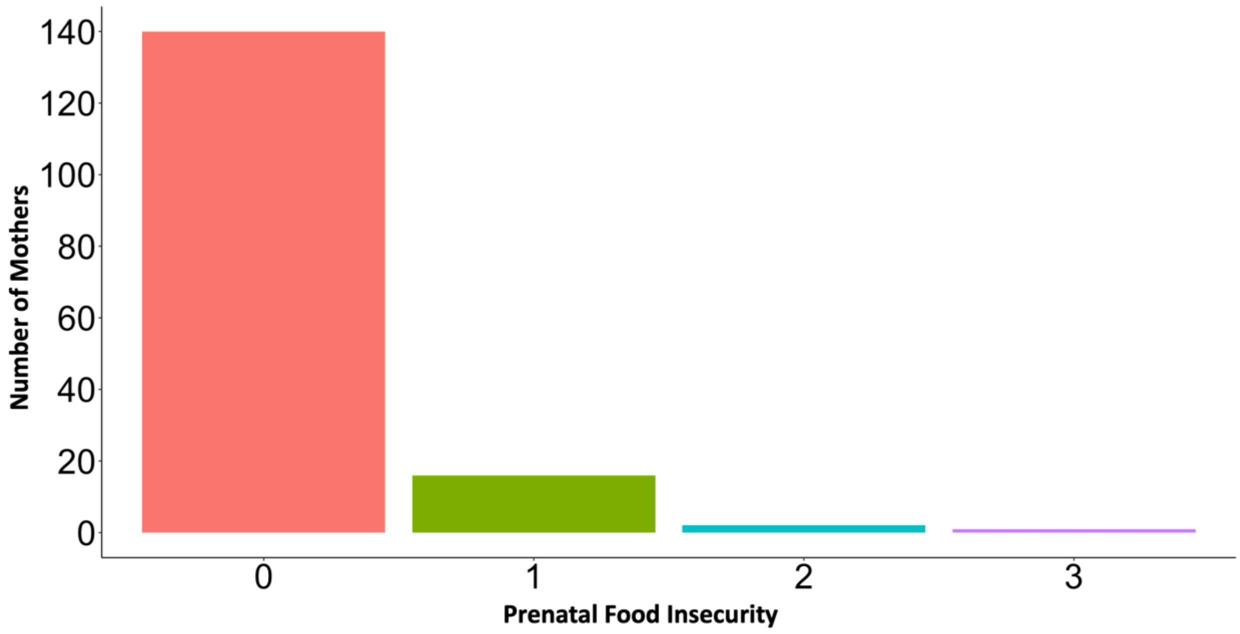

Note. Food insecurity scores could range from 0 to 3, with higher scores indicating greater experience of food insecurity in the past year.

Figure S3

Channel regions of 128-channel Hydrocel EGI net layout used in the regional electroencephalography (EEG) analyses: frontal, central, parietal, temporal, and occipital.

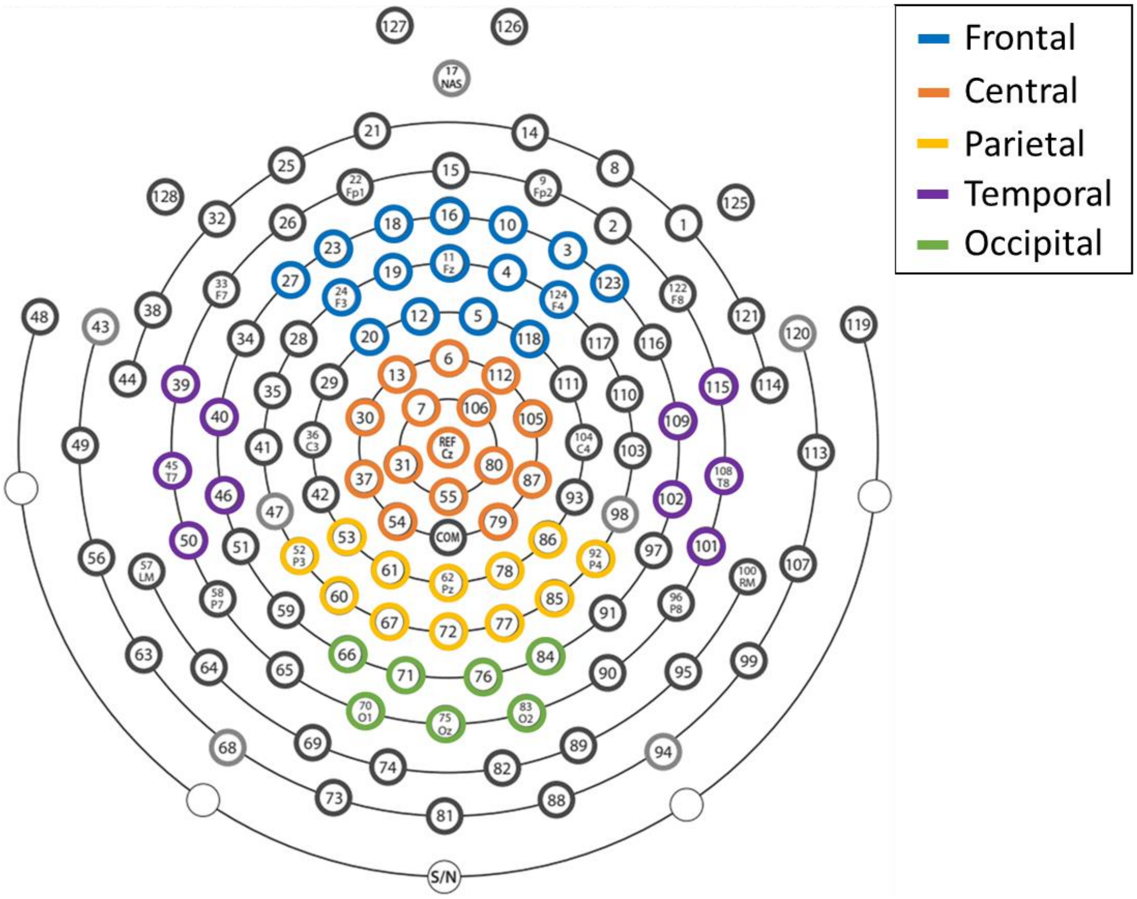

Table S1

Descriptive statistics for absolute theta, alpha, beta, and gamma power, as well as correlations between absolute EEG power and other study variables.

	Absolute Theta (log_{10}; μV^2)	Absolute Alpha (log_{10}; μV^2)	Absolute Beta (log_{10}; μV^2)	Absolute Gamma (log_{10}; μV^2)
Family Income-to-Needs (ln)	-.02	.02	.11	.16*
Parental Education (years)	-.10	-.08	.02	.09
Maternal hair cortisol (ln; pg/mg)	.03	.00	.05	.13
Maternal Perceived Stress	-.17*	-.19*	-.16*	-.10
Food Insecurity	.01	.01	-.02	-.09
Maternal Receptive Vocabulary	-.21**	-.21**	-.09	.06
Breastfed	-.08	-.03	.05	.19*
Formula Fed	-.04	-.02	-.06	-.16*
Infant Gestational Age at Birth (weeks)	.03	.10	.14	.09
Infant Weight at Birth (lbs)	.00	-.02	-.02	-.01
Infant Sex	.07	.08	.04	-.01
Infant Race	.07	.13	.04	-.06
Infant Ethnicity	-.02	-.04	-.10	-.11
Infant Age at Lab Visit (weeks)	.19*	.04	.06	.25**
Mean	0.68	0.24	0.06	0.03
SD	0.16	0.07	0.03	0.02
N	160	160	160	160

Note. Breastfed (1 = exclusively breastmilk fed, 0 = exclusively formula fed, 0 = mixed breastmilk and formula fed); Formula fed (1 = exclusively formula fed, 0 = mixed breastmilk and formula fed, 0 = exclusively breastmilk fed); Infant Sex (0 = Male, 1 = Female); Infant Race (0 = White, 1 = Non-white); Infant Ethnicity (0 = Non-Hispanic/Latino, 1 = Hispanic/Latino). Unadjusted ***p* < .01 **p* < .05.

Table S2

Results of the simultaneous multiple regressions examining associations between prenatal family income-to-needs (Model 1), parental education (Model 2) and whole-brain absolute theta, alpha, beta, and gamma power in infants.

**Model 1 (N = 151)**	**Absolute Theta**		**Absolute Alpha**		**Absolute Beta**		**Absolute Gamma**	
Predictors	β	95% CI	β	95% CI	β	95% CI	β	95% CI
Family Income-to-needs (ln)	.03	-0.11, 0.17	.07	-0.07, 0.21	.16†	0.01, 0.30	.18†	0.03, 0.33
Infant Age (weeks)	.19*	0.04, 0.34	.03	-0.12, 0.17	.04	-0.11, 0.19	.24*	0.08, 0.39
Infant Sex	.11	-0.03, 0.26	.13	-0.01, 0.28	.11	-0.03, 0.26	.05	-0.10, 0.20
Number of Epochs	-.38**	-0.54, -0.22	-.45**	-0.61, -0.30	-.50**	-0.66, -0.34	-.42**	-0.59, -0.26
Cohort	-.17	-0.32, -0.01	-.11	-0.27, 0.04	.01	-0.14, 0.17	.17	0.01, 0.34
	R^2 = .24, $F(5, 145)$ = 9.31**		R^2 = .25, $F(5, 145)$ = 9.89**		R^2 = .25, $F(5, 145)$ = 9.55**		R^2 = .23, $F(5, 145)$ = 8.54**	
**Model 2 (N = 160)**	**Absolute Theta**		**Absolute Alpha**		**Absolute Beta**		**Absolute Gamma**	
Predictors	β	95% CI	β	95% CI	β	95% CI	β	95% CI
Parental Education (years)	-.03	-0.17, 0.11	-.01	-0.15, 0.13	.08	-0.06, 0.22	.11	-0.04, 0.25
Infant Age (weeks)	.21**	0.07, 0.35	.06	-0.08, 0.20	.06	-0.08, 0.21	.23**	0.08, 0.38
Infant Sex	.11	-0.02, 0.25	.13	-0.01, 0.27	.09	-0.05, 0.24	.04	-0.11, 0.18
Number of Epochs	-.41**	-0.56, -0.26	-.45**	-0.60, -0.30	-.49**	-0.64, -0.33	-.39**	-0.54, -0.23
Cohort	-.13	-0.28, 0.03	-.10	-0.26, 0.05	.00	-0.15, 0.16	.15	0.00, 0.31
	R^2 = .26, $F(5, 154)$ = 10.97**		R^2 = .25, $F(5, 154)$ = 10.43		R^2 = .23, $F(5, 145)$ = 9.35**		R^2 = .20, $F(5, 154)$ = 7.55**	

Note. Infant Sex (0 = Male, 1 = Female); Cohort (0 = Cohort 1, 1 = Cohort 2); FDR-adjusted ***p* < .01 **p* < .05 †*p* < .07.

Table S3

Results of the simultaneous multiple regressions examining associations between prenatal family income-to-needs and whole-brain relative theta, alpha, beta, and gamma power in infants excluding infants older than two months of age (N = 14).

**Model 1 (N = 139)**	**Relative Theta**		**Relative Alpha**		**Relative Beta**		**Relative Gamma**	
Predictors	β	95%	β	95% CI	β	95% CI	β	95% CI
Family Income-to-needs (ln)	-.15†	-0.30, 0.01	.03	-0.13, 0.19	.17†	0.01, 0.33	.14†	-0.01, 0.29
Infant Age (weeks)	.16†	0.01, 0.32	-.25*	-0.41, -0.09	-.15†	-0.31, 0.01	.11	-0.04, 0.26
Infant Sex	-.08	-0.23, 0.08	.09	-0.07, 0.25	.07	-0.09, 0.23	-.02	-0.17, 0.13
Number of Epochs	.37**	0.20, 0.55	-.15	-0.33, 0.03	-.33**	-0.51, -0.15	-.33**	-0.50, -0.17
Cohort	-.10	-0.27, 0.07	-.10	-0.27, 0.07	.11	-0.06, 0.29	.27**	0.11, 0.43
	R^2 = .15, $F(5, 133)$ = 4.84**		R^2 = .12, $F(5, 133)$ = 3.68**		R^2 = .13, $F(5, 133)$ = 4.03**		R^2 = .17, $F(5, 133)$ = 5.49**	

Note. Infant Sex (0 = Male, 1 = Female); Cohort (0 = Cohort 1, 1 = Cohort 2); FDR-adjusted ***p* < .01 **p* < .05 †*p* < .10.

Table S4

Results of the simultaneous multiple regressions examining associations between prenatal family income-to-needs and whole-brain relative theta, alpha, beta, and gamma power in infants excluding families with income-to-needs ratios greater than 20 (N = 12).

**Model 1 (N = 139)**	**Relative Theta**		**Relative Alpha**		**Relative Beta**		**Relative Gamma**	
Predictors	β	95%	β	95% CI	β	95% CI	β	95% CI
Family Income-to-needs (ln)	-.17†	-0.33, -0.01	.04	-0.12, 0.20	.17†	0.02, 0.33	.16†	0.00, 0.32
Infant Age (weeks)	.06	-0.11, 0.23	-.21*	-0.38, -0.04	-.07	-0.24, 0.09	.22*	0.05, 0.38
Infant Sex	-.10	-0.26, 0.06	.10	-0.06, 0.26	.09	-0.07, 0.24	.02	-0.14, 0.18
Number of Epochs	.40**	0.23, 0.58	-.18*	-0.36, 0.00	-.38**	-0.55, -0.20	-.32**	-0.50, -0.14
Cohort	-.10	-0.28, 0.08	-.12	-0.30, 0.05	.12	-0.06, 0.29	.30**	0.12, 0.48
	$R^2 = .15$, $F(5, 133) = 4.87$**		$R^2 = .12$, $F(5, 133) = 3.64$**		$R^2 = .15$, $F(5, 133) = 4.59$**		$R^2 = .19$, $F(5, 133) = 6.24$**	

Note. Infant Sex (0 = Male, 1 = Female); Cohort (0 = Cohort 1, 1 = Cohort 2); FDR-adjusted ***p* < .01 **p* < .05 †*p* < .07.

Table S5

Results of the simultaneous multiple regressions examining associations between prenatal family income-to-needs and whole-brain relative theta, alpha, beta, and gamma power in infants without log-transforming income-to-needs ratios.

**Model 1 (N = 151)**	**Relative Theta**		**Relative Alpha**		**Relative Beta**		**Relative Gamma**	
Predictors	β	95%	β	95% CI	β	95% CI	β	95% CI
Family Income-to-needs (ln)	-.14[†]	-0.29, 0.02	.05	-0.10, 0.20	.15[†]	-0.01, 0.30	.12[†]	-0.04, 0.27
Infant Age (weeks)	.08	-0.08, 0.25	-.24**	-0.40, -0.08	-.10	-0.26, 0.06	.23**	0.07, 0.39
Infant Sex	-.10	-0.26, 0.05	.11	-0.04, 0.26	.08	-0.07, 0.24	.00	-0.16, 0.15
Number of Epochs	.39**	0.22, 0.56	-.20*	-0.37, -0.03	-.34**	-0.52, -0.17	-.29**	-0.46, -0.12
Cohort	-.11	-0.28, 0.06	-.10	-0.27, 0.07	.12	-0.05, 0.29	.27**	0.10, 0.44
	R^2 = .14, F(5, 145) = 4.65**		R^2 = .14, F(5, 145) = 4.59**		R^2 = .11, F(5, 145) = 3.70**		R^2 = .17, F(5, 145) = 6.00**	

Note. Infant Sex (0 = Male, 1 = Female); Cohort (0 = Cohort 1, 1 = Cohort 2); FDR-adjusted ***p* < .01 **p* < .05 [†]*p* < .20.

Table S6

Results of the simultaneous multiple regressions examining associations between prenatal family income-to-needs (Models 1 and 3), parental education (Models 2 and 4) and whole-brain relative and absolute theta, alpha, beta and gamma power excluding infants with outlying relative (N = 6) and absolute EEG power values (N = 5).

**Model 1 (N = 145)**	**Relative Theta**		**Relative Alpha**		**Relative Beta**		**Relative Gamma**	
Predictors	β	95% CI	β	95% CI	β	95% CI	β	95% CI
Family Income-to-needs (ln)	-.17*	-0.32, -0.02	.02	-0.13, 0.17	.19*	0.04, 0.34	.20*	0.05, 0.35
Age (weeks)	.07	-0.08, 0.23	-.23**	-0.38, -0.07	-.10	-0.25, 0.06	.25**	0.08, 0.41
Sex	-.11	-0.26, 0.04	.17	0.02, 0.31	.05	-0.11, 0.20	-.03	-0.19, 0.13
Number of Epochs	.46**	0.28, 0.63	-.28**	-0.45, -0.11	-.43**	-0.60, -0.26	-.27**	-0.44, -0.09
Cohort	-.07	-0.24, 0.10	-.11	-0.28, 0.05	.13	-0.04, 0.30	.22*	0.05, 0.40
	R^2 = .20, F(5, 139) = 6.78**		R^2 = .20, F(5, 139) = 6.88**		R^2 = .18, F(5, 139) = 5.98**		R^2 = .18, F(5, 139) = 6.15**	
**Model 2 (N = 154)**	**Relative Theta**		**Relative Alpha**		**Relative Beta**		**Relative Gamma**	
Predictors	β	95% CI	β	95% CI	β	95% CI	β	95% CI
Parental Education (years)	-.05	-0.20, 0.11	-.10	-0.26, 0.05	.11	-0.05, 0.26	.18	0.02, 0.33
Age (weeks)	.04	-0.11, 0.20	-.19*	-0.33, -0.04	-.06	-0.22, 0.09	.23*	0.08, 0.38
Sex	-.10	-0.25, 0.05	.18	0.03, 0.33	.02	-0.13, 0.17	-.04	-0.19, 0.11
Number of Epochs	.37**	0.20, 0.54	-.21*	-0.37, -0.05	-.38**	-0.55, -0.21	-.22*	-0.39, -0.06
Cohort	-.02	-0.19, 0.15	-.14	-0.30, 0.03	.08	-0.09, 0.25	.18	0.02, 0.35
	R^2 = .13, F(5, 148) = 4.41**		R^2 = .18, F(5, 148) = 6.29**		R^2 = .13, F(5, 148) = 4.43**		R^2 = .15, F(5, 148) = 5.39**	
**Model 3 (N = 146)**	**Absolute Theta**		**Absolute Alpha**		**Absolute Beta**		**Absolute Gamma**	
Predictors	β	95% CI	β	95% CI	β	95% CI	β	95% CI
Family Income-to-needs (ln)	-.02	-0.16, 0.13	.04	-0.11, 0.19	.13	-0.02, 0.28	.15	0.00, 0.31
Age (weeks)	.21*	0.06, 0.36	.04	-0.12, 0.19	.03	-0.12, 0.19	.27**	0.11, 0.43
Sex	.13	-0.02, 0.28	.15	0.00, 0.30	.10	-0.05, 0.26	.00	-0.15, 0.16
Number of Epochs	-.30**	-0.48, -0.14	-.40**	-0.58, -0.23	-.46**	-0.63, -0.28	-.36**	-0.54, -0.18
Cohort	-.16	-0.32, 0.01	-.10	-0.27, 0.06	.01	-0.15, 0.18	.20	0.03, 0.37
	R^2 = .20, F(5, 140) = 6.85**		R^2 = .20, F(5, 140) = 6.83**		R^2 = .18, F(5, 140) = 6.24**		R^2 = .20, F(5, 140) = 6.98**	
**Model 4 (N = 155)**	**Absolute Theta**		**Absolute Alpha**		**Absolute Beta**		**Absolute Gamma**	
Predictors	β	95% CI	β	95% CI	β	95% CI	β	95% CI
Parental Education (years)	-.08	-0.22, 0.07	-.06	-0.21, 0.09	.03	-0.12, 0.18	.06	-0.09, 0.21
Age (weeks)	.24**	0.09, 0.38	.07	-0.07, 0.22	.06	-0.09, 0.21	.26**	0.11, 0.41
Sex	.13	-0.02, 0.27	.14	0.00, 0.29	.08	-0.07, 0.23	-.01	-0.16, 0.14
Number of Epochs	-.35**	-0.51, -0.19	-.40**	-0.57, -0.24	-.44**	-0.61, -0.27	-.32**	-0.47, -0.14
Cohort	-.11	-0.27, 0.05	-.09	-0.25, 0.07	.00	-0.16, 0.17	.18	0.01, 0.34
	R^2 = .23, F(5, 149) = 8.76**		R^2 = .21, F(5, 149) = 7.81**		R^2 = .18, F(5, 149) = 6.37**		R^2 = .18, F(5, 149) = 6.33**	

Note. Infant Sex (0 = Male, 1 = Female); Cohort (0 = Cohort 1, 1 = Cohort 2); FDR-adjusted **p < .01 *p < .05.

Table S7

Sensitivity analyses examining the association between prenatal family income-to-needs and infant relative EEG power excluding infants with outlying relative power values.

**N = 100**	**Relative Theta**		**Relative Alpha**		**Relative Beta**		**Relative Gamma**	
Predictors	β	95% CI	β	95% CI	β	95% CI	β	95% CI
Family Income-to-needs (ln)	-.21†	-0.42, -0.01	.09	-0.13, 0.30	.20†	-0.01, 0.40	.20†	-0.01, 0.42
Parental Education (years)	.06	-0.20, 0.31	-.12	-0.38, 0.15	.04	-0.22, 0.30	.01	-0.26, 0.28
Maternal Hair Cortisol (ln; pg/mg)	-.09	-0.29, 0.11	.03	-0.18, 0.24	.11	-0.09, 0.31	.08	-0.14, 0.29
Maternal Perceived Stress	.13	-0.07, 0.33	-.17	-0.37, 0.03	-.03	-0.23, 0.17	-.02	-0.23, 0.19
Maternal Receptive Vocabulary	.09	-0.15, 0.32	-.05	-0.29, 0.19	-.02	-0.25, 0.22	-.11	-0.36, 0.13
Breastfed	-.07	-0.27, 0.14	.20	-0.01, 0.41	.00	-0.21, 0.20	-.14	-0.35, 0.07
Formula Fed	.02	-0.21, 0.26	-.02	-0.26, 0.22	.02	-0.22, 0.25	-.03	-0.28, 0.21
Infant Gestational Age at Birth (weeks)	-.27*	-0.46, -0.08	.12	-0.08, 0.31	.21*	0.02, 0.40	.28*	0.07, 0.48
Infant Weight at Birth (lbs)	.22†	0.01, 0.43	-.12	-0.34, 0.09	-.22†	-0.43, -0.01	-.14	-0.36, 0.08
Infant Sex	.06	-0.13, 0.24	.08	-0.11, 0.26	-.12	-0.30, 0.06	-.14	-0.33, 0.05
Infant Race	-.02	-0.22, 0.17	.23†	0.02, 0.43	-.04	-0.24, 0.16	-.24†	-0.45, -0.03
Infant Ethnicity	.28†	0.03, 0.54	-.13	-0.39, 0.13	-.27†	-0.52, -0.02	-.24	-0.50, 0.03
Infant Age at Lab Visit (weeks)	.06	-0.13, 0.24	-.22*	-0.41, -0.03	-.08	-0.27, 0.11	.26*	0.07, 0.46
Number of Epochs	.37**	0.17, 0.56	-.20*	-0.40, 0.00	-.34**	-0.54, -0.15	-.26*	-0.47, -0.06
Cohort	.05	-0.20, 0.29	-.14	-0.38, 0.11	-.08	-0.32, 0.16	.16	-0.09, 0.42
	$R^2 = .37$, $F(15, 84) = 3.23$**		$R^2 = .33$, $F(15, 84) = 2.80$**		$R^2 = .35$, $F(15, 84) = 3.04$**		$R^2 = .37$, $F(15, 84) = 3.34$**	

Note. Breastfed (1 = exclusively breastmilk fed, 0 = exclusively formula fed, 0 = mixed breastmilk and formula fed); Formula fed (1 = exclusively formula fed, 0 = mixed breastmilk and formula fed, 0 = exclusively breastmilk fed); Infant Sex (0 = Male, 1 = Female); Infant Race (0 = White, 1 = Non-white); Infant Ethnicity (0 = Non-Hispanic/Latino, 1 = Hispanic/Latino); Cohort (0 = Cohort 1, 1 = Cohort 2); FDR-adjusted **$p < .01$ *$p < .05$ †$p < .10$.

Comparing associations between prenatal family income, parental education, and infant whole-brain relative EEG power

As an additional robustness check, we tested whether the strength of associations between prenatal family ITN and relative EEG power were significantly different from the strength of associations between prenatal parental education and relative EEG power based on the results presented in Table 4. For these analyses, we divided the difference between standardized beta coefficients for prenatal family ITN and parental education by the difference between standard errors for prenatal family ITN and parental education (i.e., $t = (\beta_{\text{Family Income}} - \beta_{\text{Parental Education}})/(SE_{\text{Family Income}} - SE_{\text{Parental Education}})$). We did not observe significant differences in the magnitude of associations between prenatal family ITN and parental education for relative theta (effect size difference = 0.26, $t(14) = 1.59$, unadjusted $p = .12$), alpha (effect size difference = 0.18, $t(14) = 1.14$, unadjusted $p = .25$), beta (effect size difference = 0.14, $t(14) = 0.77$, unadjusted $p = .44$), or gamma power (effect size difference = 0.20, $t(14) = 1.12$, unadjusted $p = .27$) in infants.
